# GWAS*-*biologically informed SNP selection improves genomic relationship matrices and genetic parameter estimation in forest trees: insights from spruce and Eucalyptus

**DOI:** 10.3389/fpls.2026.1974694

**Published:** 2026-10-21

**Authors:** Yousry A. El-Kassaby, Eduardo P. Cappa

**Affiliations:** 1 Faculty of Forestry and Environmental Stewardship, The University of British Columbia, Vancouver, BC, Canada; 2 Instituto Nacional de Tecnología Agropecuaria (INTA), Instituto de Recursos Biológicos, Centro de Investigación en Recursos Naturales, Buenos Aires, Argentina; 3 Consejo Nacional de Investigaciones Científicas y Técnicas (CONICET), Buenos Aires, Argentina

**Keywords:** genomic pairwise kinship, GWAS, heritability, theoretical accuracy, tree breeding

## Abstract

Accurate estimation of quantitative genetic parameters is critical for genomic selection in forest tree breeding. Compared with pedigree-based relationship matrices (***A***-matrices), genomic relationship matrices (***G***-matrices) better capture realized genetic relationships, including Mendelian sampling and cryptic relatedness. This study evaluated a genome-wide association study (GWAS)-based SNP selection strategy to improve ***G***-matrix construction and estimates of additive genetic variance, heritability, and breeding values in white spruce (*Picea glauca*) and Eucalyptus (*Eucalyptus grandis*), two species with contrasting genome sizes and genotyping platforms. Progeny trials were analyzed using both pedigree-based best linear unbiased prediction (ABLUP) and genomic BLUP (GBLUP). Genomic relationship matrices were constructed from complete SNP datasets and compared with pedigree-based relationship matrices. To evaluate the impact of marker selection, SNPs were ranked according to the absolute magnitude of their GWAS-estimated effects, and sequential subsets of informative markers were used to reconstruct ***G***-matrices. Parameter estimates and theoretical accuracy from reduced SNP panels were compared with those from the complete datasets. Genomic relationship matrices generated connected family networks, revealing hidden relatedness and overcoming limitations of pedigree-based relationships. GBLUP yielded consistently higher heritability estimates for all traits in white spruce but only for tree volume in Eucalyptus. GWAS-informed SNP selection showed that marker informativeness was more important than marker number; in white spruce, ~50,000 informative SNPs performed comparably to the full 467K SNP panel for some traits, although marker requirements varied among traits. In contrast, gains in Eucalyptus were limited, likely due to reduced representation of trait-associated variation. The results demonstrate that integrating realized genomic relationship matrices with biologically informed SNP selection can provide genetic parameter estimates and theoretical accuracies comparable to those obtained with much larger marker sets while substantially reducing genotyping requirements for genomic evaluation. Responses were species- and trait-dependent. Reduced gains in Eucalyptus may reflect SNP ascertainment bias and other factors limiting the representation of causal genetic variation. Overall, biologically relevant and genomically representative markers were more important than marker density alone, and optimized SNP selection may reduce genotyping requirements while maintaining effective genomic evaluation in forest tree breeding.

## 1 Introduction

Traditional quantitative genetic analyses rely on the average numerator relationship matrix (***A***-matrix), which reflects the genealogical relationships among related individuals (resemblance among relatives). This matrix, typically derived from structured pedigrees established through mating designs (Wright, 1922), is fundamental for estimating genetic variance components and predicting individuals’ breeding values (Henderson, 1975; Gilmour et al., 1995). While this approach has been successful in achieving substantial gains in plant breeding and forestry, the long breeding cycles in trees often impede the rate of genetic improvement. The breeder’s equation (ΔG = i × r × σ_a_/L) describes the expected response to selection (ΔG) as a function of selection intensity (i), selection accuracy (r), additive genetic standard deviation (σ_a_), and breeding cycle length (L) (Lush, 1937). While the breeder’s equation offers various avenues for optimization, long breeding cycles remain a significant obstacle to tree improvement, largely due to the time needed for pedigree development and field evaluations. To overcome this, tree breeders have employed strategies such as using open-pollinated families (Stonecypher et al., 1964), pedigree reconstruction (El-Kassaby and Lstibůrek, 2009), and juvenile-mature correlations (Lambeth, 1980) to compress breeding cycles, but with varying degrees of success. Despite these approaches, reducing breeding cycle length remains one of the principal challenges in tree improvement programs.

Advances in high-throughput genotyping technologies have enabled the cost-effective generation of genome-wide single nucleotide polymorphism (SNP) data. These DNA sequences enable the accurate determination of the actual fraction of alleles shared between individuals, leading to the estimation of their realized genomic pairwise kinship (***G***-matrix) (VanRaden, 2008). This genomic relationship matrix has revolutionized quantitative genetics as it simultaneously captures both the expected relationships defined by pedigree and the realized genomic relationships inherited through both recent and distant ancestry (Powell et al., 2010). The ***G***-matrix has been successfully used to estimate genetic variance components and individual breeding values across various scenarios: pedigree-free (Porth et al., 2013a), partial pedigree (Gamal El-Dien et al., 2016, 2018), and full pedigree (Zapata-Valenzuela et al., 2013).

The growing adoption and central role of the ***G***-matrix in genomic analyses underscore the need to evaluate both the quality and quantity of SNPs used in its construction. Beyond capturing realized genealogical relationships, SNPs may also be in linkage disequilibrium (LD) with causal variants underlying traits of interest. This dual role suggests that individual SNPs contribute unequally to genomic relationship estimation and raises the possibility that trait-informative markers could generate a more biologically meaningful ***G***-matrix than one constructed from all available SNPs. Building on this concept, El-Kassaby et al. (2024) proposed constructing the ***G***-matrix using only GWAS-selected SNPs, based on the premise that trait-associated markers provide a more informative representation of the underlying genetic architecture while preserving realized genomic relationships among individuals.

Here, we extend the work of El-Kassaby et al. (2024) by evaluating the universality and robustness of GWAS-selected SNPs for constructing genomic relationship matrices across progeny trials of two forest tree species, spruce and Eucalyptus. These species differ markedly in genome size (~20 Gb vs. ~640 Mb) and were genotyped using contrasting platforms (genotyping-by-sequencing and SNP arrays, respectively), providing an opportunity to assess the broad applicability of the proposed approach across diverse genomic and experimental contexts. For each species and trait, heritability estimates and theoretical accuracies were compared between pedigree- (ABLUP) and genomic-based (GBLUP) models. In the GBLUP analyses, genomic relationship matrices were constructed using sequentially increasing subsets of SNPs, representing progressively higher marker densities, culminating in the complete set of available markers. Heritability estimates and theoretical accuracies obtained from each SNP subset were then plotted as a function of markers density, with the corresponding ABLUP estimates included as a benchmark. This framework enabled evaluation of the minimum number of informative SNPs required to achieve stable genomic parameter estimates and theoretical accuracy of breeding values comparable to, or exceeding, those obtained using the full market set and pedigree-based analyses.

## 2 Materials and methods

### 2.1 Trial description, genetic material, and evaluated traits

#### 2.1.1 Picea glauca

The *Picea glauca* genetic material used in this study originated from the D1 breeding region of the Alberta Agriculture and Forestry white spruce breeding program. Phenotypic data and tissue samples were collected from three long-term progeny trials established in Alberta, Canada: Calling Lake (CALL), Carson Lake (CARS), and Red Earth (REDE). The trials were established in 1986 (CALL) and 1987 (CARS and REDE) using a randomized complete block design with six replicates and 5- or 6-tree row plots planted at a spacing of 2.5 × 2.5 m. Detailed descriptions of the geographic locations, climatic conditions, and experimental designs of the three sites are provided in Cappa et al. (2022a; **Table 1**).

The original progeny trials comprised 150 open-pollinated families representing ten provenances. From this population, a representative subset of 80 families spanning low-, intermediate-, and high-growth classes (based on 30-year height measurements) was selected for detailed phenotypic and genomic analyses. Sampling targeted eight progeny per family at CALL and REDE and four progeny per family at CARS, yielding 1,483 individuals. An additional 142 candidate trees representing 19 families were selected through forward selection across the three trials. The final study population consisted of 1,625 individuals from 99 half-sib families.

Four traits representing growth, wood quality, and physiological performance were evaluated. Total tree height (HT, cm) and diameter at breast height (DBH, cm) were measured at age 30 years. Wood density (WD, kg m^-3^) was determined from 5-mm increment cores extracted at breast height from 35-year-old trees. Ring-by-ring density profiles were obtained using X-ray densitometry following the protocol described by Ukrainetz et al. (2008). Annual rings formed before 1995 were excluded to improve measurement reliability, and whole-tree WD was calculated by weighting annual ring density according to basal area increment.

Long-term intrinsic water-use efficiency was assessed using stable carbon isotope composition (δ^13^C). Ground wood samples obtained from the residual sections of the density cores were analyzed by continuous-flow isotope ratio mass spectrometry and normalized relative to the Vienna Pee Dee Belemnite (VPDB) international standard.

Summary statistics for all phenotypic traits are presented in Table 2 of Cappa et al. (2022a).

#### 2.1.2 Eucalyptus grandis

The *Eucalyptus grandis* genetic material used in this study originated from the fourth-generation breeding population of UPM-Forestal Oriental S.A. Phenotypic data were collected from three open-pollinated half-sib progeny trials representing two successive breeding generations: the second-generation Pandule trial and the third-generation Young and Gallinal trials. The trials were established using an incomplete block design with an alpha-lattice arrangement. The Pandule trial comprised 20 replicates and 25 incomplete blocks of single-tree plots, whereas the Young and Gallinal trials each comprised eight replicates and 10 incomplete blocks of four-tree row plots. All trials were planted at a spacing of 3.0 × 2.0 m. Detailed descriptions of the geographic locations, climatic conditions, and experimental designs are provided in **Supplementary Table S1** of Jurcic et al. (2025).

The original breeding population consisted of 549 open-pollinated families in the second-generation Pandule trial, including 209 families derived from the first breeding generation and 340 families introduced through genetic infusions. The third-generation Young and Gallinal trials comprised 126 open-pollinated families. From these populations, a representative subset was selected for detailed phenotypic and genomic analyses, including 175 families from Pandule, 40 from Young, and 88 from Gallinal. Sampling ranged from 1 to 14 progeny per family in Pandule, 7 to 27 in Young, and 3 to 10 in Gallinal, resulting in a final dataset of 1,738 individuals.

Growth and wood quality traits were assessed at approximately three years of age (3.7, 3.7, and 3.9 years for Pandule, Young, and Gallinal, respectively). Growth traits included total tree height (HT, m) and diameter at breast height (DBH, cm), which were measured directly, while stem volume (VOL, m^3^) was estimated from these measurements. Wood quality traits, namely wood density (WD, kg m^-3^) and pulp yield (PY, %), were predicted using near-infrared (NIR) spectroscopy following the methodology described by Jurcic et al. (2025). These wood quality measurements were obtained from a subset of trees that included all individuals selected for the present study.

### 2.2 Genomic data

#### 2.2.1 Picea glauca

Genotyping-by-sequencing (GBS) was performed using the EcoT22I restriction enzyme following the protocol of Chen et al. (2013). Sequence reads were aligned to the white spruce reference genome assembly (WS77111-v2), and read alignment, tag construction, and initial SNP calling were conducted as described by Cappa et al. (2022a). The resulting SNP dataset was filtered to retain biallelic markers with a maximum missing data rate of 30%, a minimum minor allele count of one, and a maximum site read depth of 70. Missing genotypes were imputed using the mean observed allele at each locus. After quality control, the final dataset comprised 1,599 individuals genotyped at 467,224 (467K) high-quality biallelic SNPs.

#### 2.2.2 Eucalyptus grandis

A total of 1,738 trees were genotyped using two SNP array platforms: 979 individuals with the EUChip60K Illumina array (Silva-Junior et al., 2015) and 759 individuals with the Axiom 72K Eucalyptus SNP array. The distribution of genotyped individuals across platforms and trials was 713 (Illumina) and 7 (Axiom) for Pandule, 547 (Axiom) for Young, and 266 (Illumina) and 205 (Axiom) for Gallinal.

Subsequent analyses were restricted to the 28,181 SNPs shared between both platforms. Markers were filtered using the R package synbreed (Wimmer et al., 2012) to retain loci with a minor allele frequency (MAF) ≥ 0.05 and a call rate (CR) ≥ 0.95. In addition, Mendelian conflicts exceeding 10% were identified and verified using qcf90 (Masuda et al., 2019) from the BLUPF90 software suite (Misztal et al., 2018). Following quality control, 19,826 high-quality SNPs were retained for subsequent analyses.

### 2.3 Pedigree correction

#### 2.3.1 Picea glauca

The pedigree of *Picea glauca* open-pollinated (OP) families was verified and corrected by comparing expected pedigree relationships with realized genomic relationships estimated from the genomic relationship matrix (***G***-matrix), following the approach proposed by Muñoz et al. (2014). After excluding 59 individuals due to parentage conflicts, pedigree records were corrected for 202 of the remaining 1,540 trees. These corrections identified five previously unrecorded maternal parents and 100 pollen donors, increasing the number of known parents from 99 to 204 (for further details, see Cappa et al., 2022a). The number of genotyped progenies per maternal parent ranged from 1 to 20 across all trials, and from 1 to 8 within individual sites.

#### 2.3.2 Eucalyptus grandis

The pedigree of *Eucalyptus grandis* OP families spanning four breeding generations was verified and corrected by comparing expected pedigree relationships with realized genomic relationships derived from the genomic relationship matrix (***G***-matrix), following the methodology described by Muñoz et al. (2014). Pairwise genomic relationship coefficients were evaluated for significant deviations from their expected theoretical values (e.g., 0.25 for half-sib relationships). Parentage assignments were subsequently reviewed and manually corrected using a custom R script.

### 2.4 Statistical analysis

To account for spatial micro-environmental variation while optimizing computational efficiency, statistical analyses were performed in a two-step framework. In the first step, individual traits were analyzed independently within each combination of site and species. We fitted pedigree-based classical design models to estimate replicate effects (for white spruce) and replicate plus incomplete block effects (for *Eucalyptus grandis*), all treated as random effects. In the second step, adjusted phenotypes were derived by subtracting the predicted design effect from the observed raw phenotypes and subsequently used as response variables in the genetic analyses (see below). In this implementation, the uncertainty associated with the adjusted phenotypes was not carried forward to the second stage, and all observations were assigned equal weight regardless of their individual precision, which may have reduced statistical efficiency relative to a single-stage analysis. Finally, all adjusted phenotypes were standardized to a mean of zero and unit variance.

These adjusted values were then modeled using a multi-site individual-tree mixed-model framework to perform both pedigree-based (ABLUP) and genomic-based (GBLUP) best linear unbiased predictions. The multi-site mixed model is expressed as:

(1) $$\left[\begin{array}{c} y_{1}\\ y_{2}\\ y_{3} \end{array}\right]=\left[\begin{array}{ccc} X_{1} & 0 & 0\\ 0 & X_{2} & 0\\ 0 & 0 & X_{3} \end{array}\right]\left[\begin{array}{c} \beta_{1}\\ \beta_{2}\\ \beta_{3} \end{array}\right]+\left[\begin{array}{ccc} Z_{a_{1}} & 0 & 0\\ 0 & Z_{a_{2}} & 0\\ 0 & 0 & Z_{a_{3}} \end{array}\right]\left[\begin{array}{c} a_{1}\\ a_{2}\\ a_{3} \end{array}\right]+\left[\begin{array}{c} e_{1}\\ e_{2}\\ e_{3} \end{array}\right]$$

where $y=\left[y_{1}^{\prime },y_{2}^{\prime },y_{3}^{\prime }\right]$ represents the vector of adjusted phenotypes across sites; $\text{β}=\left[{\text{β}}_{1}^{\prime },\;{\text{β}}_{2}^{\prime },\;{\text{β}}_{3}^{\prime }\right]$ denotes the fixed site effects; $a=\left[a_{1}^{\prime },\;a_{2}^{\prime },\;a_{3}^{\prime }\right]$ is the random additive genetic vector, and $e=\left[e_{1}^{\prime },\;e_{2}^{\prime },\;e_{3}^{\prime }\right]$ is the residual vector. The symbol (′) indicates the transpose operation. Additive genetic effects were assumed to follow $a∼N\left(0,G_{0}⊗A\right)$ for ABLUP, where ***G***_0_ is an unstructured genetic covariance matrix and ***A*** is the pedigree relationship matrix. We assumed an unstructured (co)variance matrix for the genetic effects (***G***_0_). Residuals were modeled as $e∼N\left(0,R_{0}⊗I\right)$ where ***R***_0_ is a 3 × 3 residual covariance matrices. Given that the sites were assessed separately, the residual covariance across sites is assumed to be zero. For the GBLUP analyses, the pedigree-based matrix ***A*** was replaced with the genomic relationship ***G***, constructed according to VanRaden (2008):


$$G=\frac{WW^{\prime }}{2\sum_{k}p_{k}\left(1-\;p_{k}\right)}$$

where, ***W*** is the centered matrix of SNP covariates, and *p_k_* is the current (or observed) allele frequency of the genotyped trees for marker *k*.

### 2.5 Heritability estimates

Narrow-sense individual heritability estimates across sites were estimated for each *i^th^* trait and species ( ${\hat{h}}_{i}^{2}$) under both pedigree- and genomic-based frameworks as follows:


$${\hat{h}}_{i}^{2}=\frac{{\hat{\sigma }}_{a_{i}}^{2}}{\left({\hat{\sigma }}_{a_{i}}^{2}+{\hat{\sigma }}_{e_{i}}^{2}\right)}$$

where ${\hat{\sigma }}_{a_{i}}^{2}$ is the estimated genetic variance for the *i^th^* trait, and ${\hat{\sigma }}_{e_{i}}^{2}$ is the estimated residual variance for the *i^th^* trait from the multi-site model (Equation 1).

All variance components and heritability parameters were estimated in R using the remlf90 function within the “breedR” package (Muñoz and Sanchez, 2020). The estimation procedure utilized the Expectation-Maximization (EM) algorithm, followed by an Average Information (AI) step to compute standard errors for both variance components and heritability estimates (Chateigner et al., 2020). This R-package implementation is based on the REMLF90 and AIREMLF90 programs from the BLUPF90 suite (Misztal et al., 2018). Finally, marker effects were derived using the postGSF90 program from the same BLUPF90 software family (Aguilar et al., 2019).

### 2.6 Theoretical accuracy

To evaluate and compare the performance of the different pedigree- and genomic-based methods, we also calculated the theoretical accuracy (TA) of the predicted additive genetic values. For each tree *i*, the TA was computed across all traits and prediction models (ABLUP and GBLUP) as follows:


$${\text{TA}}_{i}=\sqrt{1-\frac{{\text{PEV}}_{i}}{{\hat{\sigma }}_{a}^{2}}}$$

where PEV*_i_* (prediction error variance) represents the prediction error variance of the *i*-th individual, obtained directly from the corresponding diagonal element of the inverted coefficient matrix of the mixed model equations (MME).

### 2.7 SNP selection protocol

To evaluate how marker density influences genetic parameter estimation, we examined the impact of SNP-marker number on both genomic-based narrow-sense heritability and the theoretical accuracies of breeding values using the multi-site model (Equation 1). To prevent data circularity, the GWAS-based SNP selection protocol was subsequently performed after partitioning the population of each tree species into training (~30%) and validation (~70%) datasets, while strictly preserving the original experimental design structure, including family and site representation. The training dataset was used exclusively to estimate SNP effects, whereas the validation dataset was reserved for the construction of genomic relationship matrices and subsequent genomic analyses. Marker selection was determined by ranking the absolute values of SNP effects from largest to smallest, with SNP effects averaged across all study sites. These effects were derived via a multi-site single-step genome-wide association study (ssGWAS) framework using the training dataset (Aguilar et al., 2019). **Supplementary Table S1** provides the estimated marker effects for all 467,224 SNPs in white spruce and 19,826 SNPs in Eucalyptus across all evaluated traits.

To evaluate the impact of marker density, nested subsets of 1,000, 10,000, 50,000, 100,000, 200,000, 300,000, and 400,000 SNPs for white spruce, and 2,478, 4,956, 7,435, 9,913, 12,391, 14,870, and 17,348 SNPs for *E. grandis*, were selected from the total available panel (467,224 and 19,826 SNPs, respectively). These specific marker subsets were then utilized to construct the corresponding genomic additive relationship matrices for individuals in the validation dataset.

## 3 Results and discussion

As expected, genomic-based pedigree networks produced complete connectedness among families and their offspring across all progeny trials in both species, in contrast to the fragmented networks derived from conventional pedigree records (**Figure 1**). This enhanced connectedness demonstrates the ability of genomic information to recover contemporary and ancestral genetic relationships that are missing, hidden, or incorrectly represented in traditional pedigrees. By leveraging realized genomic relationships, reconstructed pedigrees revealed previously unknown genealogical links among families and identified pedigree errors commonly encountered in forest tree breeding programs (Adams et al., 1988; Devey et al., 2002). The resulting networks therefore provided a more complete and biologically meaningful representation of the breeding populations.

**Figure 1:**
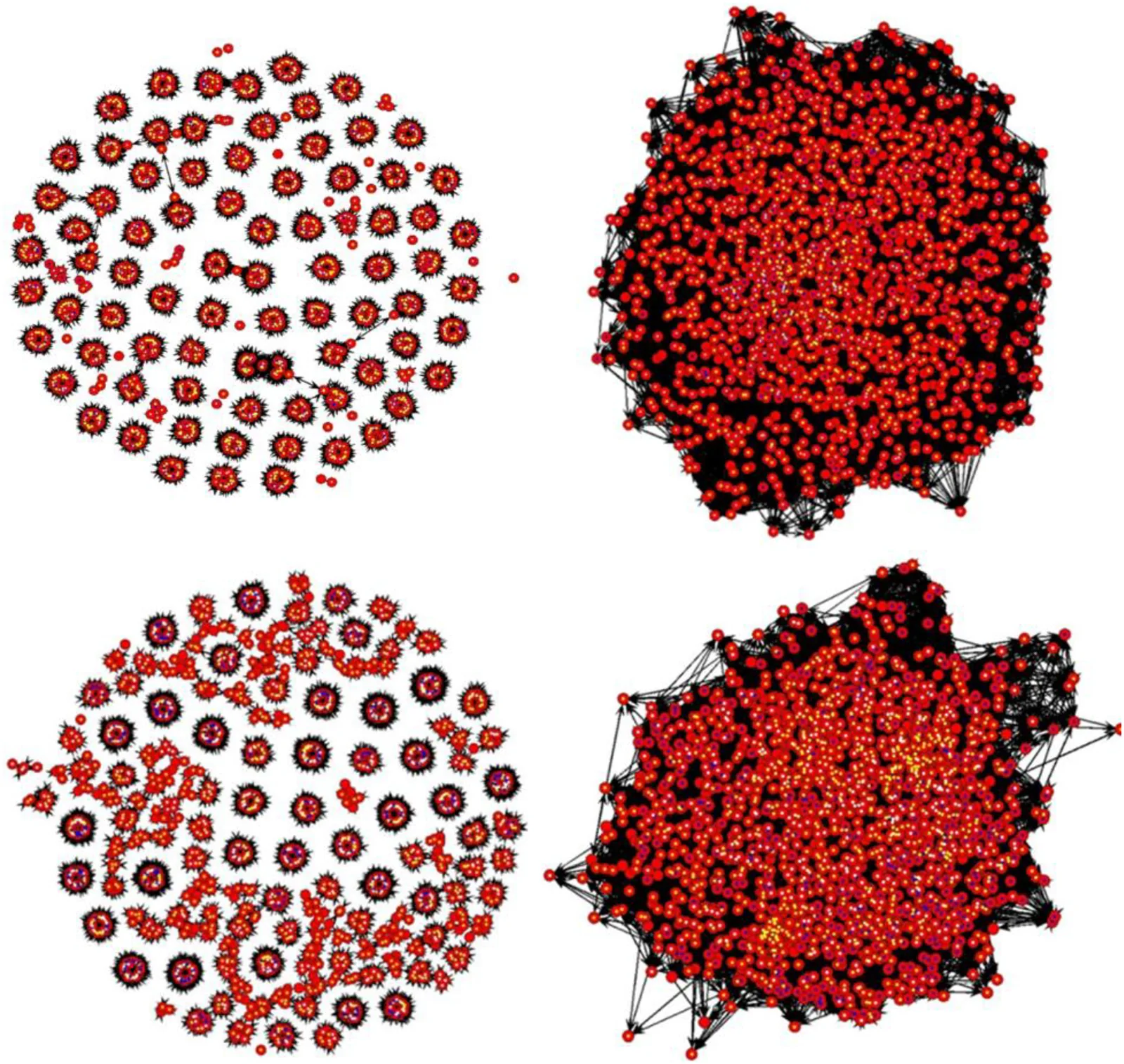
Pedigree (left)- and genomic (right)-based networks with a minimal genomic pairwise kinship threshold set at 0.05 for white spruce (top) and eucalypts (bottom).

Improved pedigree connectedness has important implications for quantitative genetic analyses. Greater connectivity among families improves the partitioning of genetic and environmental sources of variation, resulting in more accurate estimates of additive genetic variance and heritability (Gamal El-Dien et al., 2016, 2018; El-Kassaby et al., 2024). It also enhances breeding value prediction by increasing the flow of genetic information across families, progeny trials, and generations while reducing biases associated with incomplete or erroneous pedigree records. Consequently, integrating contemporary and ancestral genomic relationships provides a more robust framework for genetic evaluation, enabling breeding programs to maximize the value of the information already present within existing populations without the need for additional controlled crosses or the lengthy process of developing structured pedigrees.

Using the complete SNP panels to construct genomic relationship matrices (***G***-matrices), GBLUP-derived heritability estimates were compared with those obtained from ABLUP (***A***-matrices) across both species (**Table 1**). In white spruce, GBLUP consistently produced higher heritability estimates than ABLUP for all four evaluated traits, indicating that genomic relationships captured additional additive genetic variation beyond that represented by the recorded pedigree. Conversely, Eucalyptus showed a different pattern. Except for tree volume, GBLUP produced lower heritability estimates than ABLUP, suggesting that the available SNP array captured less of the realized genomic variation associated with these traits. This difference likely reflects variation in marker informativeness and genomic representation between the two genotyping strategies, with the GBS-derived dataset in white spruce providing broader representation of genome-wide relationships than the fixed SNP array used in Eucalyptus.

**Table 1:** Average cross-site heritability estimates (with standard errors) for white spruce and Eucalyptus traits in the validation population.

Species	White spruce			Eucalyptus	
Trait	ABLUP	GBLUP	Trait	ABLUP	GBLUP
Height	0.64 (0.26)	0.78 (0.19)	Volume	0.43 (0.15)	0.43 (0.16)
DBH	0.42 (0.14)	0.58 (0.39)	Wood density	0.65 (0.08)	0.47 (0.17)
Wood density	0.62 (0.18)	0.69 (0.25)	Pulp yield	0.61 (0.22)	0.51 (0.17)
δ^13^C	0.61 (0.26)	0.77 (0.12)			

White spruce (N = 1,063) was evaluated for height, diameter at breast height (DBH), wood density, and water-use efficiency (δ^13^C). Eucalyptus (N = 1,080) was assessed for tree volume, wood density, and pulp yield. Heritability estimates were derived using both ABLUP and GBLUP methods, highlighting the difference between pedigree- and genomic-based networks on genetic parameter estimation.

To further assess the influence of marker informativeness on variance components, heritability estimates, and theorical accuracy of breeding values, GBLUP models based on increasing numbers of GWAS-selected SNPs were compared with ABLUP and full-panel GBLUP models. In white spruce, increasing the number of GWAS-selected SNPs consistently resulted in progressively larger additive genetic variance and heritability estimates, while simultaneously increasing the theoretical accuracy of breeding values. As the number of markers increased, GBLUP surpassed ABLUP and eventually matched or exceeded the estimates obtained with the complete SNP panel (467K; **Figure 2A**; **Supplementary Table S2**). Notably, approximately 50K informative SNPs were sufficient to achieve theoretical accuracies and genetic parameter estimates comparable to, or greater than, that obtained with the full marker dataset, particularly for HT and δ^13^C. This result indicates that a relatively small subset of informative markers can provide an effective representation of genomic relationships, thereby reducing marker redundancy without compromising the consistency of genetic parameters estimates relative to the full marker panel. The rapid decline in absolute marker effects observed with increasing SNP number (**Figure 3A**) further supports the limited contribution of additional markers beyond this point.

**Figure 2:**
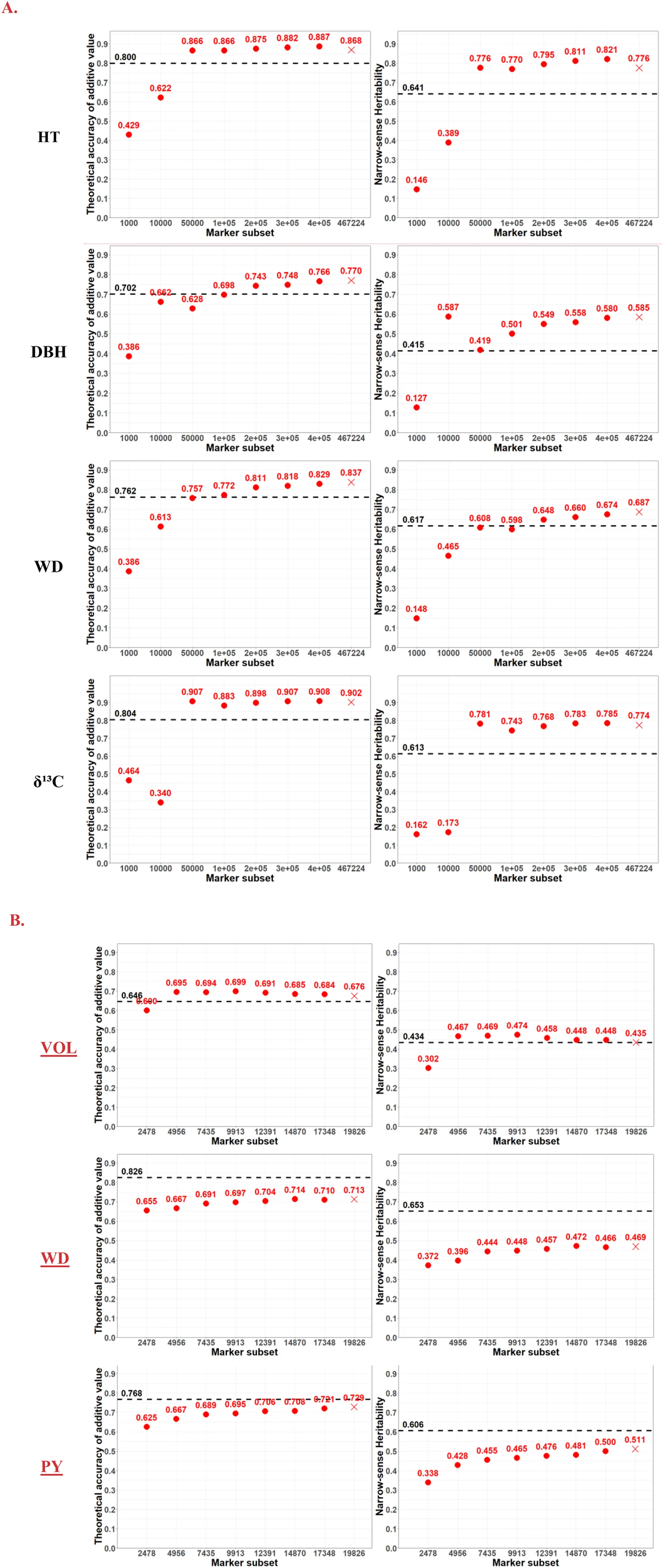
Theoretical accuracy of the predicted additive genetic values (left column) and narrow-sense heritability (right column). **(A)** White spruce (height (HT), diameter at breast height (DBH), wood density (WD), and water-use efficiency (δ^13^C, stable carbon isotope ratio)). **(B)** Eucalyptus (volume (VOL), wood density (WD), and pulp yield (PY)). Estimates are shown for an increasing number of SNPs, ranging from 1,000 to 467,224 for white spruce and from 2,478 to 19,826 for Eucalyptus. Red dots represent SNPs subsets selected according to the absolute GWAS effect sizes (ranked from largest to smallest) in the additive genomic-based GBLUP model. Dashed black horizontal lines indicate the theoretical accuracy and heritability estimates obtained from the conventional pedigree-based ABLUP model. Red “x” marks denote the estimates from the genomic-based GBLUP model using the full set of 467,224 SNPs for white spruce and 19,826 SNPs for Eucalyptus. Corresponding numerical values are displayed above the red dots (GWAS-selected GBLUP), red “x” marks (Full SNP panel GBLUP), and dashed lines (ABLUP).

**Figure 3:**
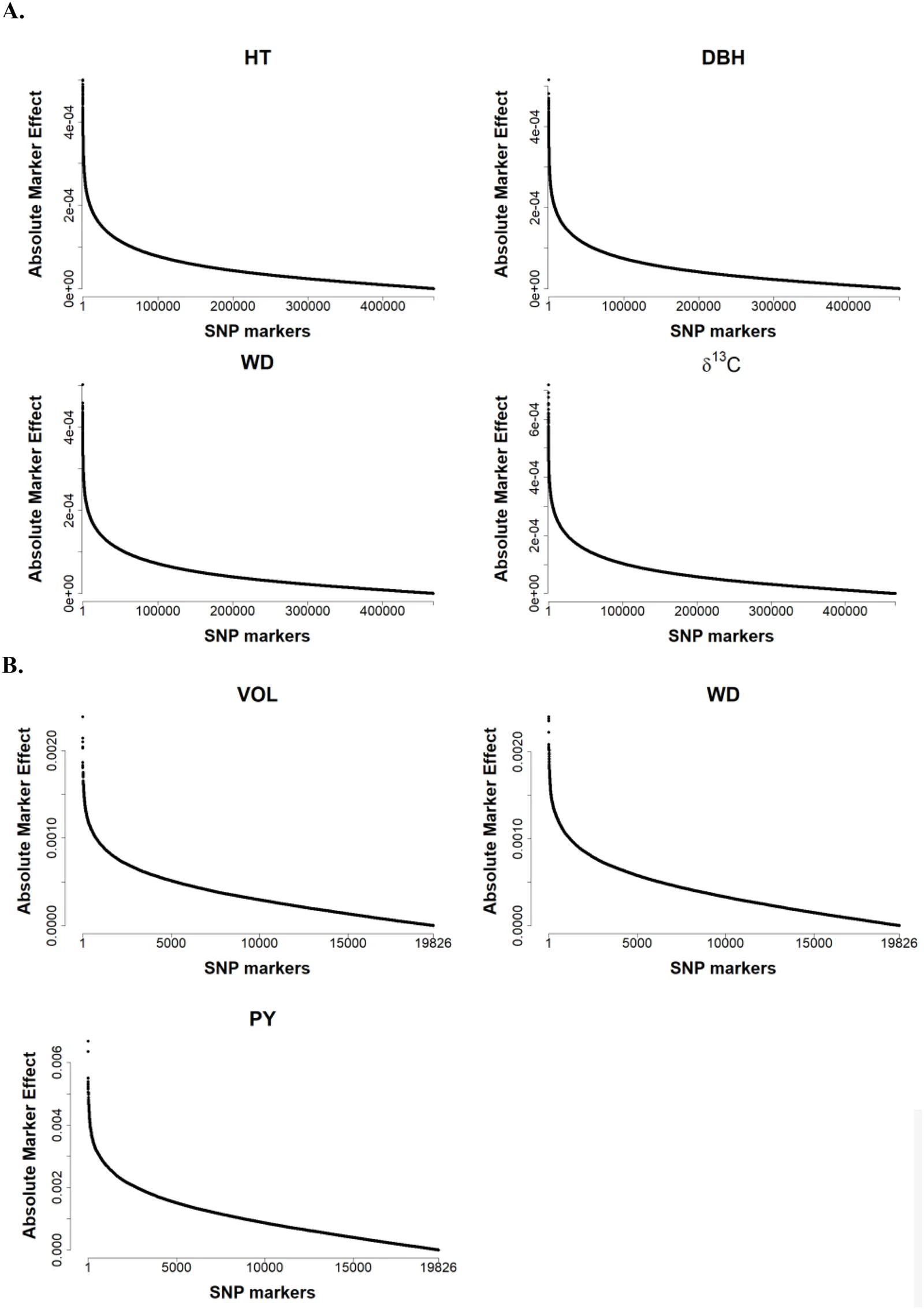
Estimated marker absolute effects arranged in descending order. **(A)** White spruce (height (HT), diameter at breast height (DBH), wood density (WD), and water-use efficiency (δ^13^C, stable carbon isotope ratio)) and **(B)** Eucalyptus (volume (VOL), wood density (WD), and pulp yield (PY)).

Eucalyptus showed a contrasting response to SNP selection, despite exhibiting a greater concentration of large-effect SNPs among the top-ranked markers (**Figure 3B**). With the exception of tree volume, ABLUP consistently produced higher heritability estimates and theoretical accuracy of breeding values than GBLUP, regardless of the number of SNPs included in the genomic relationship matrix (**Figure 2B**; **Supplementary Table S2**). This suggests that the EuChip60K Illumina and 72K Eucalyptus Axiom arrays did not adequately capture the genomic variation underlying wood density and pulp yield, although they appeared more effective for tree volume. The reduced gains observed in Eucalyptus may reflect a combination of factors, including SNP ascertainment bias and other species-specific biological and experimental characteristics. For example, ascertainment bias may occur when the SNPs represented on an array do not fully reflect the distribution of causal variants or functional genomic regions (Altshuler et al., 2000; Clark et al., 2005). Thus, the present results suggest that high marker density alone does not necessarily guarantee improved theoretical accuracy or higher heritability estimates, particularly when marker subsets differ in their ability to capture the underlying genetic relationships relevant to the target traits.

The contrasting responses of the two species indicate that the performance variance component estimates (or functions of them) and theoretical accuracies depends not simply on the number of markers, but on their informativeness and ability to capture relationships between individuals that are relevant to the genetic variation underlying the traits. For complex polygenic traits influenced by many loci with small effects (Fisher, 1918), conventional GWAS identifies only a subset of markers associated with phenotypic variation (Tam et al., 2019). Moreover, testing markers individually does not account for the interconnected pathways and gene networks through which multiple loci contribute to complex traits (Porth et al., 2013b). The GWAS-based selection strategy evaluated here provides a complementary approach by sequentially selecting SNPs according to the magnitude of their estimated ssGWAS effects rather than relying solely on individual-marker statistical significance. The resulting ranked SNP subsets were subsequently used to construct genomic relationship matrices of increasing marker density. Consequently, markers can be retained based on their estimated contribution to the trait-variation, potentially improving the ability of the resulting ***G***-matrix to capture trait-relevant genetic relationships. GWAS-informed genomic relationship matrices may provide improved representation of the genetic architecture underlying a focal trait, particularly when the trait deviates from a purely polygenic architecture, which can influence heritability and theoretical accuracy estimates relative to conventional genome-wide ***G***-matrices. This was particularly evident in white spruce, where HT and δ^13^C achieved estimates comparable to those obtained with the full 467K-marker dataset using approximately 50K selected SNPs, whereas DBH and WD required larger marker subsets to approach full-panel estimates.

Marker preselection and pruning have previously been explored in forest tree breeding using different criteria, including significant GWAS associations (Chen et al., 2023; Tan and Ingvarsson, 2022), maximization of co-ancestry resolution (Cappa et al., 2022b), random marker subsets (Chen et al., 2018, 2023; Calleja-Rodriguez et al., 2020), genomic position (Tan et al., 2017), and linkage group structure (Lenz et al., 2017). Most of these approaches prioritize association significance, marker distribution, or the resolution of genomic relationships. In contrast, the present strategy focuses on selecting markers according to the magnitude of their ssGWAS-estimated effects and evaluating their utility for genomic relationship matrix construction and genomic parameter estimation, providing a complementary criterion for identifying a compact and informative SNP panel.

The effectiveness of this approach ultimately depends on sufficient LD between the selected SNPs and the causal variants underlying the traits. This consideration is particularly important in open-pollinated species, where rapid LD decay requires adequate marker density to maintain associations with causal loci. In Eucalyptus, the available SNP arrays provide relatively dense genomic coverage, with approximately one SNP every 12–20 kb (Silva-Junior et al., 2015). For white spruce, however, the physical coverage of the SNP dataset cannot be reliably quantified because its available genome assembly is highly fragmented, comprising approximately 6.7 million contigs and 4.9 million scaffolds (Birol et al., 2013). Therefore, direct assessment of the physical distribution of the selected SNPs relative to the white spruce genome is not currently possible. Nevertheless, the sharp decline in absolute marker effects after approximately 50K SNPs in white spruce (**Figure 3A**), together with the tendency of genetic parameter estimates to stabilize as marker number increased (**Figure 2A**), provides indirect evidence that additional markers contributed relatively little information beyond this point. In Eucalyptus, the corresponding trends generally did not reach a clear plateau, except for tree volume, suggesting that the available marker panels may not have captured the underlying LD structure equally well across traits.

Importantly, these findings demonstrate that genome size and nominal marker density are insufficient predictors of variance component estimates and the theoretical accuracy of breeding values. Although the relatively small Eucalyptus genome (~640 Mb) was extensively represented by the EuChip60K and 72K arrays, with approximately 90% genome coverage and one SNP every 12–20 kb, GBLUP did not consistently outperform ABLUP. In contrast, the much larger white spruce genome (~20 Gb) yielded consistently higher estimates with a GBS-derived marker dataset when informative SNPs were selectively retained. However, these contrasting outcomes should not be attributed solely to differences in genome size and marker density. The two species also differ in SNP quality-control and filtering procedures, breeding history, generation composition, trial design, phenotyping protocols, and trait assessment age, all of which may have contributed to the observed patterns. Therefore, this comparison should be viewed as evidence that the proposed approach can be effective across markedly different genomic and breeding contexts, rather than as a direct assessment of the effects of genome size or marker platform. This comparison suggests that the ability of marker subsets to capture realized genetic relationships, rather than their absolute number or nominal physical coverage, may be an important determinant of the effectiveness of genomic relationship matrices, although this interpretation should be viewed with caution given the many biological and experimental differences between the two species.

The contrasting heritability estimates obtained from the ***A***- and ***G***-matrices for the two species are intriguing. In white spruce, ***G***-matrix-based heritability estimates consistently exceeded those derived from the ***A***-matrix across all four traits, whereas no consistent pattern was observed in Eucalyptus. It is important to note that conventional pedigree-based heritability estimates are often inflated when open-pollinated families are assumed to consist of half-sibs, as this assumption frequently does not hold in practice (**Figure 1**) (Namkoong, 1966; Squillace, 1974; Askew and El-Kassaby, 1994). This observed contrast provide insight into the commonly reported phenomenon of “missing heritability,” in which heritability estimates differ when based on conventional pedigree information versus genomic relationship matrices. Although missing heritability is influenced by numerous biological and methodological factors (see Manolio et al., 2009, for a review), our results suggest that, in Eucalyptus, the discrepancy may largely reflect statistical limitations. Specifically, heritability may be overestimated when using an inaccurate pedigree-based relationship matrix, while simultaneously underestimated when using a less informative genomic relationship matrix. The combined effects of these opposing biases likely contribute to the observed differences in PEV-based theoretical accuracy and the apparent missing heritability among the Eucalyptus traits.

Overall, the results demonstrate that GWAS-based SNP selection can provide an efficient strategy for constructing realized genomic relationship matrices and estimating quantitative genetic parameters. The substantially reduced SNP set required in white spruce illustrates the potential to achieve genetic parameter estimates and theoretical accuracies comparable to those obtained with much larger marker sets, while reducing marker redundancy and computational demands. However, the contrasting results between white spruce and Eucalyptus also indicate that the effectiveness of marker selection is trait- and population-dependent and is influenced by LD structure, marker informativeness, and the underlying genetic architecture as well as other biological, genomic, and experimental factors. Accordingly, the proposed approach should be viewed as a complementary strategy for optimizing genomic evaluations rather than as a universal replacement for high-density SNP panels. When effective, such marker optimization may facilitate more efficient genomic evaluation and contribute to accelerated genetic gain in forest tree breeding programs.

## 4 Conclusions

Overall, the genomic pairwise kinship matrix (***G***-matrix) provided a more accurate representation of genetic relationships than the pedigree-based approach in forest tree breeding populations. GWAS-informed SNP selection proved particularly effective in white spruce, where approximately 50K informative SNPs achieved PEV-based theoretical accuracies comparable to that those obtained with the full 467K-marker dataset. However, the effectiveness of SNP selection varied between species (white spruce and Eucalyptus) and among traits, indicating that marker requirements are both species- and trait-dependent. Because the two species differ in several biological, genomic, and experimental characteristics, including breeding history, trait assessment, genotyping platforms, and SNP quality-control and filtering strategies, the factors underlying these differences cannot be disentangled in the present study. Nevertheless, the results suggest that marker informativeness, may play an important role in determining theoretical accuracy beyond marker number alone. These findings demonstrate that optimized SNP selection can substantially reduce genotyping requirements while maintaining the theoretical accuracy of breeding values, thereby providing efficient framework for genomic evaluation and potentially accelerating genetic gain in forest tree breeding programs.

## Data Availability

The white spruce genotyping-by-sequencing (GBS) raw sequence data analyzed in this study are publicly available through the NCBI Sequence Read Archive (SRA) under BioProject accession PRJNA748443 (https://www.ncbi.nlm.nih.gov/bioproject/PRJNA748443). Information on the field trials, including pedigree records and adjusted, standardized phenotypic data, is available in the GitHub repository: https://github.com/RESFOR/quantitative_genetics_R/blob/e067422f5e56ec7bb98e4265e60e875603bf51b5/White_Spruce_Phenotype_Pedigree_PLoS2022.TXT. The Eucalyptus datasets supporting the conclusions of this article are available from the corresponding author upon reasonable request and without undue reservation. Requests for access to these data should be directed to EPC (cappa.eduardo@inta.gob.ar).
