# Supplementary material for: GWAS-biologically informed SNP selection improves genomic relationship matrices and genetic parameter estimation in forest trees: insights from spruce and Eucalyptus
Source: Front Plant Sci. 2026 Oct 21;17:1974694. doi: 10.3389/fpls.2026.1974694 (PMC13732133; doi:10.3389/fpls.2026.1974694)
Supplement: Supplementary file 1 [file DataSheet1.pdf]

Supplementary material

Table S2. Mean additive genetic variance (Additive), residual variance (Residual), and narrow-sense heritability (Heritability), with associated standard errors (SE), and theoretical accuracy of the predicted additive genetic values (Accuracy) estimated from the different marker subsets for spruce traits (height (HT), diameter at breast height (DBH), wood density (WD), and water-use efficiency (C13, stable carbon isotope ratio)) and Eucalyptus traits (volume (VOL), wood density (WD), and pulp yield (PY)). Values are averaged across sites.

Trait	Marker subset	Additive	Residual	Heritability	Accuracy
**White Spruce**					
HT	1,000	0.139 (0.056)	0.803 (0.093)	0.146 (0.060)	0.429
HT	10,000	0.340 (0.204)	0.542 (0.241)	0.389 (0.254)	0.622
HT	50,000	0.631 (0.252)	0.191 (0.285)	0.776 (0.375)	0.866
HT	100,000	0.627 (0.264)	0.195 (0.301)	0.770 (0.387)	0.866
HT	200,000	0.643 (0.271)	0.174 (0.311)	0.795 (0.417)	0.875
HT	300,000	0.653 (0.273)	0.160 (0.315)	0.811 (0.427)	0.882
HT	400,000	0.659 (0.099)	0.151 (0.093)	0.821 (0.110)	0.887
HT	467,224	0.630 (0.143)	0.188 (0.157)	0.776 (0.191)	0.868
DBH	1,000	0.120 (0.118)	0.831 (0.154)	0.127 (0.129)	0.386
DBH	10,000	0.495 (0.103)	0.371 (0.107)	0.587 (0.115)	0.662
DBH	50,000	0.363 (0.275)	0.528 (0.325)	0.419 (0.327)	0.628
DBH	100,000	0.431 (0.287)	0.446 (0.338)	0.501 (0.355)	0.698
DBH	200,000	0.466 (0.298)	0.403 (0.350)	0.549 (0.375)	0.743
DBH	300,000	0.474 (0.302)	0.392 (0.355)	0.558 (0.383)	0.748
DBH	400,000	0.491 (0.304)	0.373 (0.358)	0.580 (0.392)	0.766
DBH	467,224	0.496 (0.306)	0.368 (0.360)	0.585 (0.393)	0.770
WD	1,000	0.132 (0.055)	0.759 (0.091)	0.148 (0.063)	0.386
WD	10,000	0.376 (0.117)	0.446 (0.138)	0.465 (0.157)	0.613
WD	50,000	0.486 (0.125)	0.316 (0.143)	0.608 (0.170)	0.757
WD	100,000	0.479 (0.136)	0.322 (0.158)	0.598 (0.184)	0.772
WD	200,000	0.514 (0.129)	0.279 (0.149)	0.648 (0.176)	0.811
WD	300,000	0.522 (0.135)	0.268 (0.156)	0.660 (0.187)	0.818
WD	400,000	0.532 (0.132)	0.256 (0.152)	0.674 (0.183)	0.829
WD	467,224	0.541 (0.177)	0.246 (0.205)	0.687 (0.248)	0.837
C13	1,000	0.157 (0.036)	0.809 (0.083)	0.162 (0.038)	0.464
C13	10,000	0.181 (0.115)	0.832 (0.149)	0.173 (0.114)	0.340
C13	50,000	0.672 (0.067)	0.200 (0.088)	0.781 (0.083)	0.907
C13	100,000	0.629 (0.086)	0.226 (0.109)	0.743 (0.112)	0.883
C13	200,000	0.645 (0.086)	0.202 (0.109)	0.768 (0.115)	0.898
C13	300,000	0.655 (0.074)	0.190 (0.098)	0.783 (0.101)	0.907
C13	400,000	0.656 (0.082)	0.188 (0.106)	0.785 (0.112)	0.908
C13	467,224	0.649 (0.088)	0.197 (0.112)	0.774 (0.120)	0.902
**Eucalyptus**					
VOL	2,478	0.187 (0.083)	0.428 (0.08)	0.302 (0.125)	0.600
VOL	4,956	0.293 (0.082)	0.327 (0.075)	0.467 (0.116)	0.695
VOL	7,435	0.294 (0.086)	0.325 (0.078)	0.469 (0.122)	0.694

VOL	9,913	0.295 (0.106)	0.322 (0.095)	0.474 (0.154)	0.699
VOL	12,391	0.283 (0.107)	0.333 (0.097)	0.458 (0.157)	0.691
VOL	14,870	0.276 (0.108)	0.339 (0.099)	0.448 (0.159)	0.685
VOL	17,348	0.276 (0.109)	0.339 (0.100)	0.448 (0.160)	0.684
VOL	19,826	0.267 (0.109)	0.348 (0.101)	0.435 (0.161)	0.676
WD	2,478	0.292 (0.108)	0.495 (0.101)	0.372 (0.128)	0.655
WD	4,956	0.31 (0.120)	0.478 (0.112)	0.396 (0.144)	0.667
WD	7,435	0.349 (0.131)	0.442 (0.119)	0.444 (0.154)	0.691
WD	9,913	0.353 (0.134)	0.439 (0.122)	0.448 (0.158)	0.697
WD	12,391	0.362 (0.135)	0.432 (0.122)	0.457 (0.159)	0.704
WD	14,870	0.374 (0.138)	0.422 (0.124)	0.472 (0.161)	0.714
WD	17,348	0.369 (0.140)	0.427 (0.126)	0.466 (0.164)	0.710
WD	19,826	0.372 (0.142)	0.425 (0.127)	0.469 (0.166)	0.713
PY	2,478	0.335 (0.073)	0.633 (0.082)	0.338 (0.069)	0.625
PY	4,956	0.429 (0.156)	0.542 (0.141)	0.428 (0.146)	0.667
PY	7,435	0.454 (0.166)	0.517 (0.149)	0.455 (0.156)	0.689
PY	9,913	0.466 (0.170)	0.508 (0.152)	0.465 (0.159)	0.695
PY	12,391	0.477 (0.085)	0.499 (0.080)	0.476 (0.073)	0.706
PY	14,870	0.482 (0.173)	0.495 (0.154)	0.481 (0.161)	0.708
PY	17,348	0.501 (0.176)	0.477 (0.156)	0.500 (0.163)	0.721
PY	19,826	0.513 (0.179)	0.467 (0.158)	0.511 (0.165)	0.729
